# Micro-CT screening of old shell collections helps to understand the distribution of viviparity in the highly diversified clausiliid clade of land snails

**DOI:** 10.1038/s41598-019-56674-7

**Published:** 2020-01-09

**Authors:** Anna Sulikowska-Drozd, Piotr Duda, Katarzyna Janiszewska

**Affiliations:** 1 University of Lodz, Department of Invertebrate Zoology and Hydrobiology, Banacha Str. 12/16, 90-237 Lodz, Poland; 2 University of Silesia in Katowice, Faculty of Science and Technology, Będzińska Str. 39, 41-200 Sosnowiec, Poland; 3 Institute of Paleobiology, Polish Academy of Sciences, Twarda 51/55, 00-818 Warsaw, Poland

**Keywords:** Developmental biology, Evolution, Zoology

## Abstract

Current zoological research may benefit in many ways from the study of old collections of shells. These collections may provide materials for the verification of broad zoogeographical and ecological hypotheses on the reproduction of molluscs, as they include records from many areas where sampling is currently impossible or very difficult due to political circumstances. In the present paper we present data on viviparous and embryo-retention reproductive modes in clausiliid land snails (subfamily Phaedusinae) acquired from specimens collected since the nineteenth century in the Pontic, Hyrcanian, and East and Southeast Asian regions. X-ray imaging (micro-CT) enabled relatively quick screening of more than 1,000 individuals classified within 141 taxa, among which we discovered 205 shells containing embryos or eggs. Gravid individuals were found to belong to 55 species, representing, for some of these species, the first indication of brooding reproductive strategy.

## Introduction

**Figure 1:**
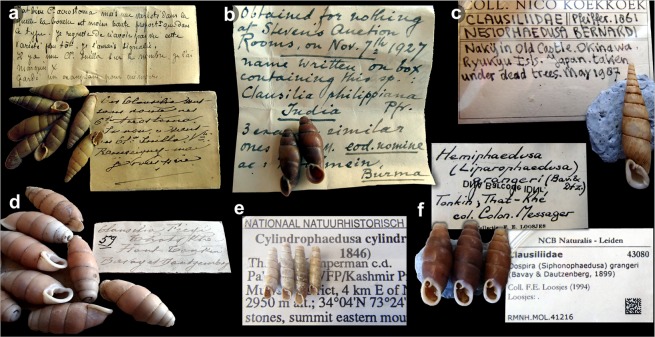
Examples of clausiliid shells and original labels from the collections of Muséum national d’Histoire naturelle, Paris; Museum of Comparative Zoology, Harvard University, Cambridge, Massachusetts; and Naturalis Biodiversity Center, Leiden: (**a**) *Synprosphyma acrostoma*, (**b**) *Oospira philippiana*, (**c**) *Luchuphaedusa bernardii*, (**d**) *Liparophaedusa freyi*, (**e**) *Cylindrophaedusa cylindrica*, (**f**) *Liparophaedusa grangeri.*

Specimens deposited in natural history museums possess huge potential as an almost limitless biodiversity resource. These preserved animals form a basis for fundamental insights into zoological systematics, biogeography, ecology, and evolution. In the past decade, collection-based research has incorporated several technological and methodological advances which have extended the scope of study far beyond alpha taxonomy and taxonomic reviews. Indisputably, the major source of input in the field has been molecular methods, including next-generation sequencing and barcoding^1,2^. Important additions to knowledge have been made possible by means of isotopic studies, geometric morphometry, and microcomputed tomography (e.g.^3–6^). Moreover, the digitisation of collections, in tandem with species modelling, has enhanced our ability to synthesise biogeographical data and our knowledge of the possible responses of species to ongoing climatic or anthropogenic threats^7,8^. In the present paper, we explore the potential of old malacological collections (Fig. 1) as a source of data for life history studies, including reproductive modes and fecundity. We focus on brooding strategy (the development of embryos within the parental reproductive system) in terrestrial gastropods of the family Clausiliidae. For this purpose, micro-CT scanning was adopted as a screening technique. We also evaluated the usefulness of dry- and ethanol-preserved specimens for this kind of research.

Among terrestrial pulmonate molluscs, several reproductive modes are recognised, among which **oviparity** (whereby laid eggs contain only a single-celled zygote or the earliest cleavage stages) seems to be the most common^9–11^. However, in a great number of species classified within at least 30 families, developing embryos are retained in the parental reproductive tract for various parts of the embryonic period^9–11^. If the period of embryo retention is very long and hatching occurs in the reproductive tract, the reproductive mode should be classified as live-bearing reproduction, or **viviparity**^12^. Formerly, the term *ovoviviparity* was frequently used for this strategy in malacological literature^9,11,13–15^, despite critical comments from researchers in other fields^16^. An intermediate strategy between oviparity and viviparity, whereby the first part of embryonic development takes place in the genital tract and the second in the ambient environment, is also widespread. This reproductive mode is referred to here as **embryo retention**. Strategies involving the development of embryos inside the parental organism, including both embryo retention and viviparity, are referred to in this paper as **brooding**. During embryo retention, nutrients and calcium for the developing offspring can be derived either from the yolk alone (lecithotrophy) or directly from the parent (matrotrophy); the latter has been proven only in a limited number of land snail taxa^17,18^.

The strategy of brooding is usually seen as an adaptation which contributes to reducing the level of mortality caused by drought or predators, as well as enhancing competition for food over the young of oviparous animals^19,20^. Brooding may have evolved in land snails in response to several different selective pressures, such as irregular onset of the rainy season in subtropics, short growing periods in temperate areas, and lack of suitable oviposition sites in habitats with extreme environmental conditions (e.g. exposed rocks)^19,20^. None of these hypotheses has been tested within a phylogenetic framework (e.g. a gastropod family). In our opinion, such analyses are hindered mainly by the lack of reliable data on the distribution of reproductive modes across taxa, geographical areas, and habitats.

Clausiliidae, probably the largest family of land snails^21^, has become a model group for studies of diversity in breeding biology in these animals (e.g.^14,22,23^). Within relatively well-studied species from Europe, several reproductive modes were identified based on long-term breeding experiments and field observations^14,23–28^. For embryo-retaining and viviparous species, it was shown that during the reproductive season (usually late spring/summer in Central Europe) a substantial proportion of adult snails are gravid, with one to several embryos in the genital tract^23,25,29,30^. Embryos are initially enveloped in the egg membrane, which contains calcium carbonate crystals; prior to parturition, an embryo may develop an embryonic shell with 1–3 whorls. The period of internal development lasts from several days to ca 3 weeks; however, the source of nutrient and calcium provision for embryos has not yet been studied. Embryos found in the genital tract constitute direct evidence of a non-oviparous reproductive strategy (i.e. brooding). In contrast, a typically oviparous species lays eggs within hours of fertilisation; thus it is unusual to find eggs or egg shells in their genital tracts during dissection^9^.

**Figure 2:**
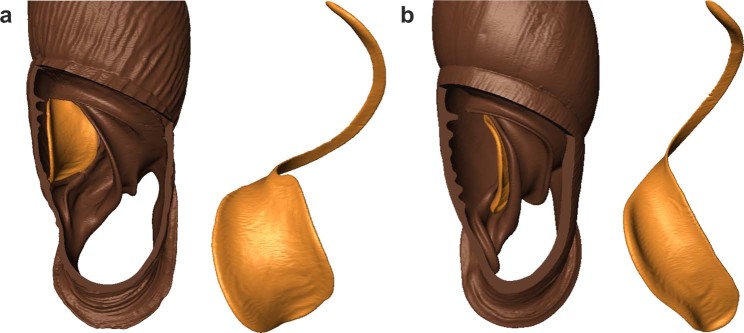
Visualisation of clausiliid shells (dorsal view of ultimate whorl with palatal wall opened and clausilium) based on micro-CT scanning: (**a**) *Euphaedusa fusaniana*, (**b**) *Oospira formosensis*. The broad clausilium plate (**a**) is correlated with brooding strategy, while the narrow clausilium (**b**) occurs only in oviparous species^13^.

Rather than conducting time-consuming experiments and field observations, we used shells from museum collections to look for embryo-retaining and viviparous taxa within Clausiliidae (subfamily Phaedusinae). As far as we know, this idea was put forward for the first time by F. E. Loosjes, who provided a foundation for studying the diversity of reproductive modes in clausiliids of East and Southeast Asia^31–33^. The genital anatomy and reproduction of Phaedusinae species were also mentioned in several papers (e.g.^34–36^); however, more detailed studies are scarce^37–39^. The correct identification of reproductive modes in Phaedusinae appears highly significant, as researchers formerly linked life history traits with taxonomically sound characters^13,40^, or traced them in the molecular phylogeny of this group^15^. The correlation between shell characters and reproductive strategy in all clausiliids is indisputable, as the delivery of a shell embryo requires the reduction or rearrangement of complex apertural barriers that occur within the ultimate whorl of the shell^41^. These apertural barriers are believed to provide protection from small predators which enter the shell through the aperture^42^; on the other hand, they may impose constraints on reproduction, mandating the laying of hard-shelled eggs and viviparity^43^. The co-existence of viviparity and certain shell characters (e.g. a broad clausilium plate) have been demonstrated for a number of Phaedusinae genera (Table 1, Fig. 2). Oviparous snails require no adaptations to enlarge the capacity of the shell channel, and may be characterised by a narrow clausilium plate^13^.

**Table 1:** Published data on reproductive strategies in Phaedusini.

Genus	reproduction mode correlated shell characters: *shape of lower lamella*/*shape of clausilium plate*	Species with proven embryo-retention or viviparity
*Phaedusa*	spirally ascending/broad	*corticina, japonica, paviei, phongthoensis, valida*,
*Euphaedusa*	spirally ascending/broad	*aculus, cetivora, digonoptyx, kurodai, porphyrea, stearnsi, tau*
*Reinia*	spirally ascending/broad	*ashizuriensis, eastlakeana, variegata*,
*Renschiphaedusa*	spirally ascending/broad	*cumingiana, recondita*
*Metazaptyx*	spirally ascending/broad	—
*Paraphaedusa*	spirally ascending/broad	—
*Parazaptyx*	spirally ascending/broad	—

The morphology of apertural barriers in the following genera has been recognised as a possible adaptation for the delivery of shelled neonates^13^. Evidence for embryo retention has been published for selected species only^13,31,53,54,56–58^.

On the other hand, previous molecular studies on clausiliids revealed that a complex system of apertural barriers, previously recognised as taxonomically significant, has evolved several times in parallel and may have even undergone reversals^15,44,45^.

**Figure 3:**
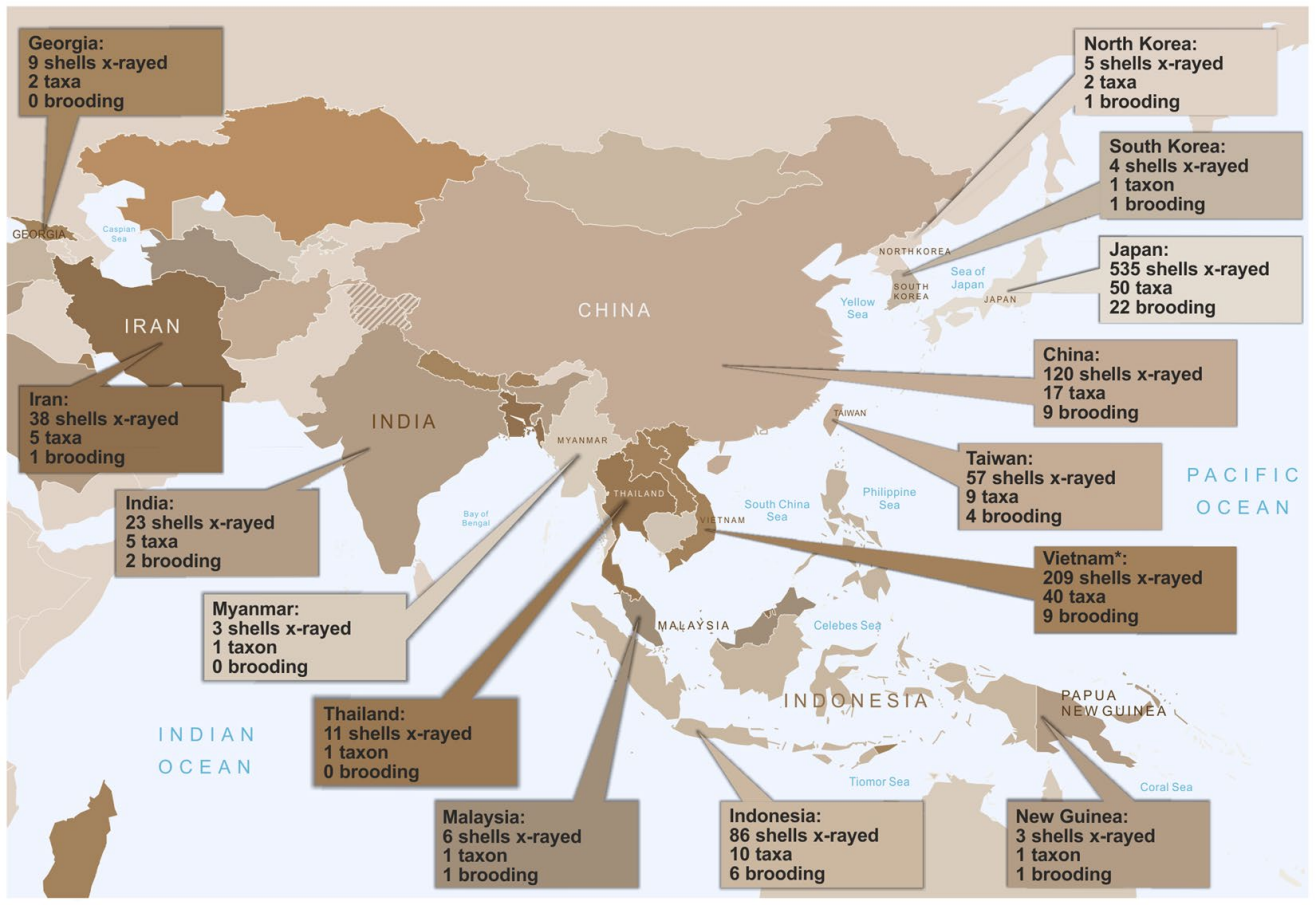
Original distribution of the Phaedusinae samples investigated in this project. For each territory, the number of individuals X-rayed, the number of taxa identified, and the number of brooding taxa have been provided. More details on shell collections and preservation technique can be found in the Supplementary Table (Table S1). *Including specimens collected in the former French colony of Indochina; no data specifying localities with greater precision are available. The base map (world borders) was obtained from United Nations Map No. 4170 Rev. 17 February 2019.

The Phaedusinae subfamily comprises approximately 500 extant species^46–48^, currently classified into 3 tribes: Phaedusini and Synprosphymini, distributed in East and Southeast Asia, and Serrulinini, inhabiting the Pontic and Hyrcanian regions of Asia^49,50^. Access to substantial parts of their distribution ranges has been cut off for political reasons within the last 80 years, but these areas had been sampled previously and abundant collections have been preserved in natural history museums across Europe and the USA (Fig. 3). These collections provide an opportunity to analyse reproductive modes in Phaedusinae without new field sampling, assuming that snails were collected alive and preserved in ethanol or dried without the removal of soft tissue.

The primary goal of the present paper was to test X-ray screening as a method for studying the reproductive strategies of gastropods kept in museum collections, both ethanol- and dry-preserved. Secondarily, as an introduction to a larger project focusing on the evolution of reproductive modes in land snails, we aimed to reassess the distribution of embryo retention and viviparity across the species-rich group Clausiliidae (Phaedusinae).

## Material and Methods

The material included shells on loan from the following museum collections and individual collectors:

- MNHN (Museum National d’Histoire Naturelle, Paris, France; dry shells, mostly from the territory formerly known as French Indochina, among them the nineteenth-century collections of A. Bavay, P. Dautzenberg, and L. G. M. Messager);
- MCZ (malacology collection; Museum of Comparative Zoology, Harvard University, Cambridge, USA; dry shells, mostly from Japan, among them the collection of Y. Hirase);
- NHM (Natural History Museum, London, UK; alcohol-preserved specimens collected in Vietnam and China);
- NBC (Naturalis Biodiversity Center, Leiden, the Netherlands; dry- and alcohol-preserved shells, among them samples from Indonesia from the collection of F. Loosjes);
- MIIZ (Muzeum i Instytut Zoologii PAN, Warsaw, Poland; alcohol-preserved shells from China, Myanmar, and Thailand);
- the private collections of M. Szekeres, E. Stworzewicz, W. Maassen, and A. Drozd.

Altogether, more than 1,000 Phaedusinae shells belonging to 141 taxa (138 species and three subspecies) were analysed (Fig. 3). The majority of material belonged to the species-rich tribe Phaedusini, whereas Serrulinini and Synprosphymini were represented by seven species each. The majority of shells (86%) had been stored dry; the rest were preserved in 70–96% ethanol.

For the sake of the consistency of the project, classification into genera after Nordsieck^46^ has been used throughout the text.

For shell examination, the non-destructive method of microcomputed X-ray tomography (micro-CT) was adopted. This technique enables the internal calcareous structure (e.g. embryonic shell) to be examined without destruction of specimens, and thus can be adapted to the study of valuable museum collections^51^. First, as a screening procedure, a single X-ray projection of each shell, generated by a micro-CT scanner, was obtained. This step alone enabled confirmation of the brooding reproduction mode and indication of the number and size of embryos. Second, gravid individuals were selected for acquisition of a series of X-ray images; subsequently, these projections were used to create three-dimensional models of internal structures of shells.

Depending on temporal availability, the following micro-CT scanners were used to acquire X-ray projections:

- Skyscan 1172 micro-CT, Naturalis Biodiversity Center, Leiden, the Netherlands;
- Zeiss Xradia MicroXCT-200, Institute of Paleobiology, Polish Academy of Sciences, Warsaw, Poland;
- GE Phoenix v|tome|x s, X-ray Microtomography Lab, Faculty of Science and Technology, University of Silesia, Katowice, Poland.

The samples illustrated in the present paper were scanned at the University of Silesia using the following scanner settings: voltage, 90–200 kV; electric current, 100–180 µA; timing, 250 ms; 1000 projections; rotation angle, 360°; voxel size, 10–20 µm, depending on shell size. Finally, the projections were used to reconstruct 3D images of shell interiors with embryos. The 3D models were created in cooperation with Custom Medical Implants Laboratory in Łódź, equipped with Amira software (v. 5.5.0), FEI Visualization Sciences Group, USA, and Geomagic Studio 2014, 3D Systems, USA, and in cooperation with the Institute of Paleobiology, PAS, in Warsaw, equipped with Avizo 7.0 Fire Edition software.

## Results

**Figure 4:**
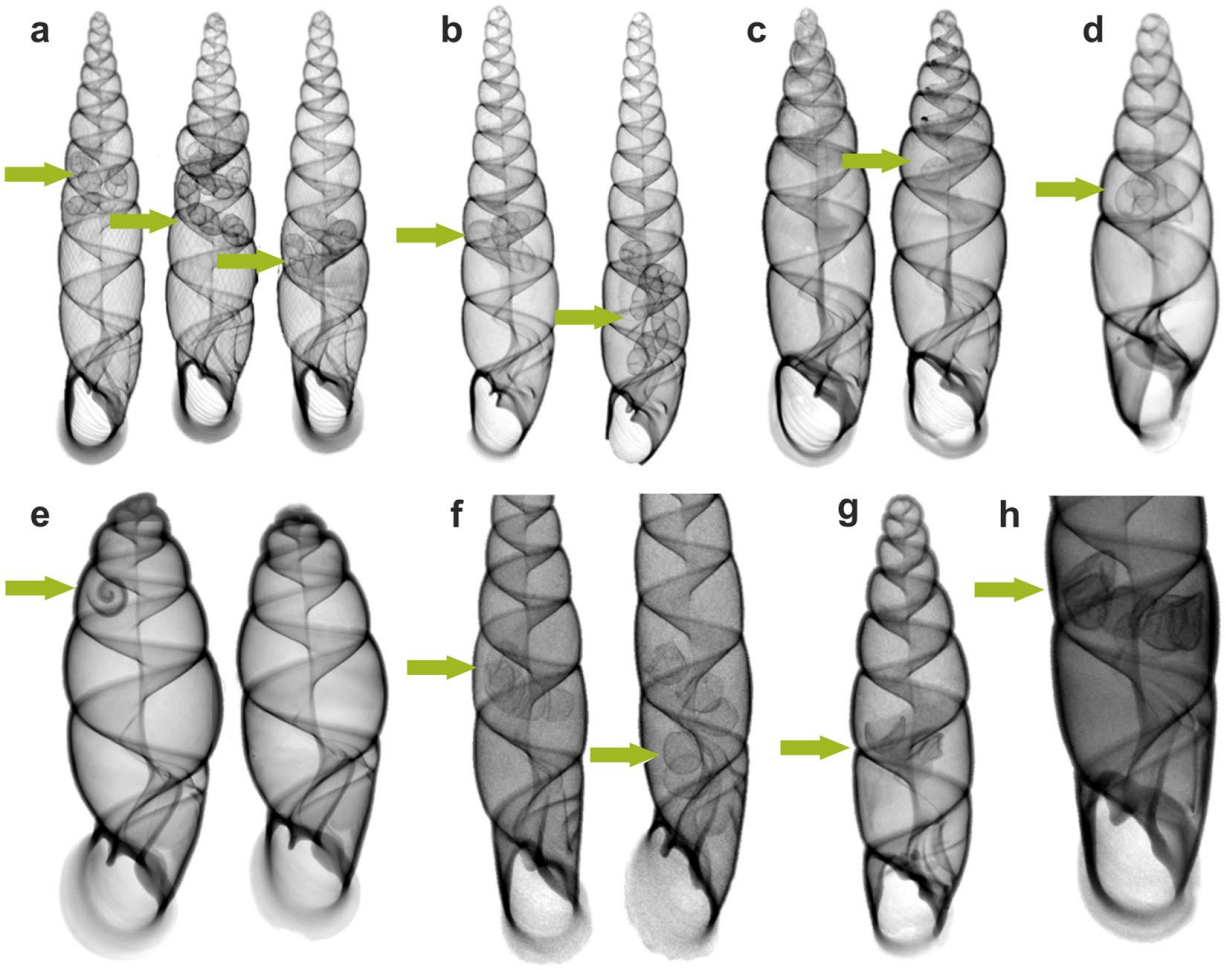
The X-ray screening of the collections enabled rapid identification of shells with eggs or embryos inside; single X-ray projections of selected species: (**a**) *Phaedusa hilberi*, (**b**) *Euphaedusa anceyi*, (**c**) *Reinia hungerfordiana*, (**d**) *Metazaptyx daemonorum*, (**e**) *Oospira philippiana*, (**f**) *O. javana*, (**g**) *Zaptyx hirasei*, (**h**) *Hemiphaedusa exilis*. Arrows indicate the *in-situ* position of embryos and egg shells. Gravid individuals derive from dry museum collections, with the exceptions of (**f**,**h**) (ethanol-preserved samples).

The use of X-ray imaging enabled relatively rapid screening of over a thousand specimens from old collections, as well as the identification of 205 shells containing embryos or egg shells (Fig. 4; Supplementary Material), constituting 19% of X-rayed shells. In dry material, the proportion of gravid snails was 18% (169/936), whereas in alcohol-preserved samples the proportion was 23% (36/155). The difference between the two types of preservation is not significant (χ^2^, p > 0.05).

Gravid individuals (with embryos or eggs) occurred in 55 species (45 containing embryos, 10 containing egg shells).

**Figure 5:**
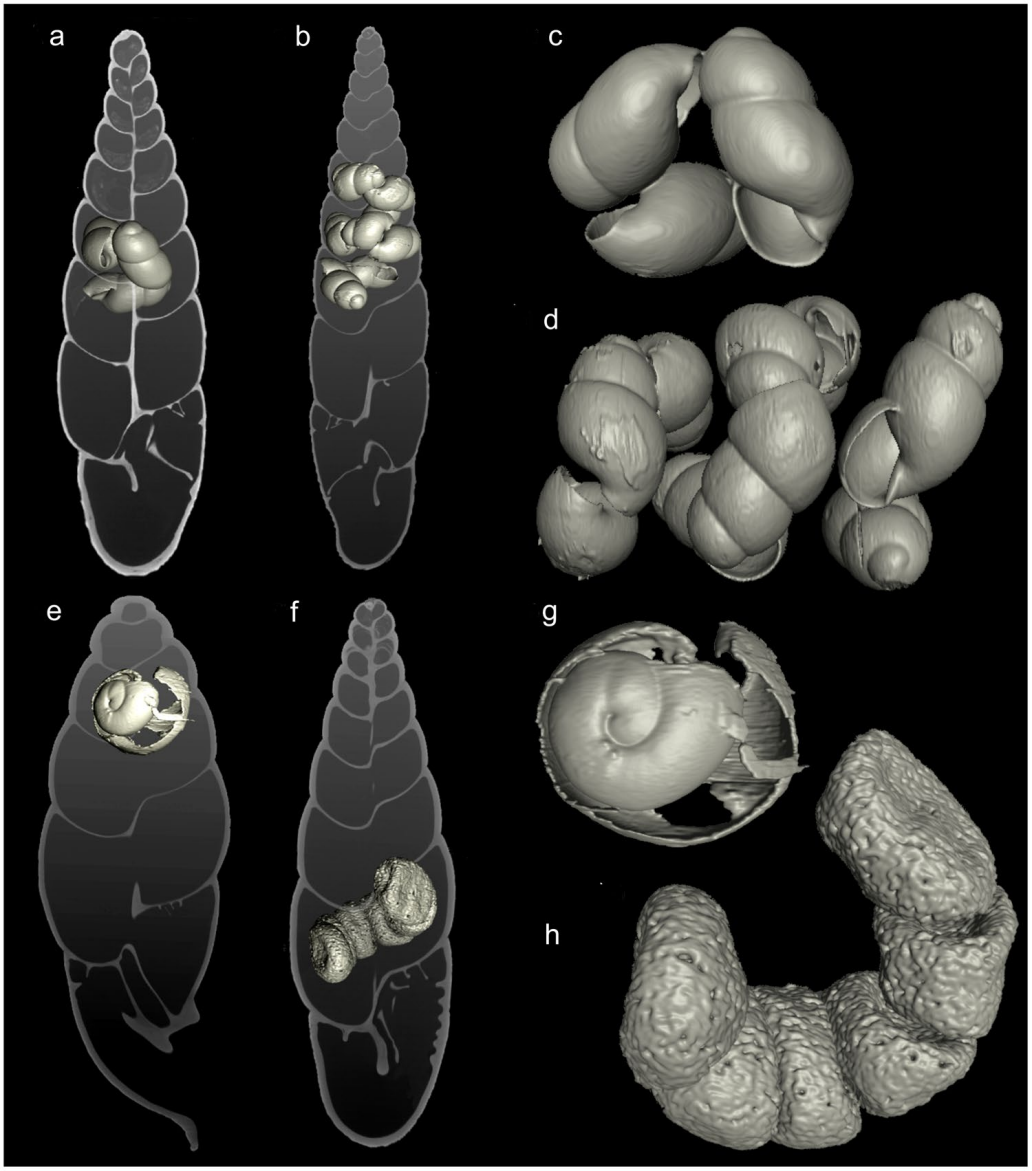
Visualisation of embryos and eggs found in clausiliid shells from museum collections based on micro-CT scanning: (**a,c**) *Phaedusa hilberi*, (**b**,**d**) *Euphaedusa fusaniana*, (**e**,**g**) *Oospira philippiana*, (**f**,**h**) *O. formosensis*. Individuals derive from dry museum collections, with the exceptions of (**f**,**h**) (ethanol-preserved samples).

Embryos were regularly found in species classified within the East and Southeast Asian genera *Phaedusa*, *Euphaedusa*, *Reinia*, *Paraphaedusa*, *Renschiphaedusa*, *Parazaptyx*, and *Metazaptyx*, albeit not in all examined individuals (Table 2). The number of embryos ranged from 1 to 12 per individual (usually from 1 to 5) (Fig. 4a–d); the size of embryonic shells reached 1.5–3 whorls (Fig. 5c,d). The greatest numbers of embryos were recorded for *Euphaedusa aculus* (12), *E. digonoptyx* (9), and *Phaedusa hilberi* (9). None of the species classified within the Synprosphymini or Ponto-Caspian Serrulinini tribes retained shelled embryos.

**Table 2:** Distribution of gravid individuals among Phaedusini samples included in the museum collections studied herein.

Genus	No. of individuals X-rayed	No. of species analysed	No. of individuals with embryos	No. of species with embryos
*Phaedusa*	186	20	72	14
*Euphaedusa*	143	16	53	15
*Reinia*	116	7	30	6
*Renchiphaedusa*	33	2	10	2
*Metazaptyx*	29	3	9	3
*Paraphaedusa*	9	4	6	3
*Parazaptyx*	7	1	3	1

Only genera with recognised morphological adaptations for brooding strategy^13^ have been included.

Selected species of other genera, e.g. *Oospira javana*, *O. miranda*, *O. ferruginea*, *O. formosensis*, *O. philippiana*, *Hemiphaedusa exilis*, and *H. ooi*, contained shelled embryos (Fig. 4e) or egg membranes with calcium crystals (Figs. 4f,h and 5f,h). Our findings proved that these species are capable of retaining fertilised eggs for at least a short time. Among them, *O. miranda* and *O. philippiana* (Fig. 5e,g) kept embryos with embryonic shells consisting of more than one whorl. Additionally, we recorded egg membranes/egg shells in *Zaptyx dolichoptyx*, *Z. hirasei* (Fig. 4g), *Z. kikaiensis*, *Stereozaptyx exulans*, and *Serrulina sieversi*. For these species, the evaluation of acquired images was not straightforward, as the eggs appeared to be unshaped or damaged, perhaps due to the conservation method.

## Discussion

The X-ray screening method adopted in this study enabled the verification of information concerning embryo retention in many snail taxa. The examined material comprises about 30% of identified Phaedusinae species, and thus should enable the reassessment of the distribution of reproductive modes within this subfamily. However, as we lack a complete phylogenetic framework for the studied group^15,49^, our conclusions are very cautious. Previously, the taxonomic system of the subfamily was based on shell characters^13,52–54^, some of which are probably related to the reproduction mode and its mechanical constraints. The significance of the reproductive mode in the relevant systematics was expressed explicitly by Nordsieck^13^, who recognised two tribes, Megalophaedusini and Phaedusini, with the latter characterised by synapomorphies correlated with ovoviviparity (a spirally ascending inferior lamella and a broad clausilium plate). The genera *Phaedusa*, *Parazaptyx*, *Metazaptyx*, *Reinia*, *Renschiphaedusa*, *Paraphaedusa*, and *Euphaedusa*, listed as members of Phaedusini^13^, were thus regarded as ovoviviparous. Brooding reproduction in these taxa was assumed based on the shape of the clausilial apparatus^13^, although findings of embryos have not been documented in all these species (Tables 1 and 2). Contrastingly, genera classified within Megalophaedusini were characterised by a steeply ascending inferior lamella and a narrow clausilium plate in common (Fig. 2). Later, the systematics of Phaedusinae were rearranged: most taxa were classified into Phaedusini, whereas some members of the former Megalophaedusini group were moved to a new tribe, Synprosphymini^46^. The problematic relationships within Phaedusinae were only partially resolved by the molecular phylogeny of the entire Clausiliidae family^49^, which showed that East and Southeast Asian Phaedusinae (only 6 species included) formed a single lineage with Ponto-Caspian Serrulininae, which had previously been regarded as a separate subfamily^46^. The recent molecular phylogeny of Japanese taxa also suggests that the Phaedusinae classification requires profound taxonomical revision^15^.

The X-ray screening of collections from East and Southeast Asia resulted in the finding of embryo-retaining species among taxa classified within *Oospira* and *Hemiphaedusa* (Figs. 4e,f,h and 5e–h) and previously attributed to oviparous Megalophaedusini^13^. Thus, the classification of Phaedusinae species into ovoviviparous and oviparous genera cannot be sustained. The presence of retained eggs in the uterus of *O. javana*^32^ also constituted evidence against this division, although the cited author, having observed egg-laying in his laboratory culture, finally classified the species as oviparous.

In a recent paper on the molecular phylogeny of Japanese clausiliids, the significance of reproductive adaptation in taxonomy was upheld^15^. The cited authors classified the species into two types of reproduction, ovoviviparity and oviparity. Among six main lineages of Japanese clausiliids, two include only oviparous taxa, two only ovoviviparous taxa; the remaining two include species employing both strategies. Altogether, ovoviviparity was indicated for 77 terminal tips (identified as 47 taxa), oviparity for 184 tips (128 taxa); 12 tips (10 taxa) were left with no reproductive strategy being attributed to them on the tree provided^15^. Our study overlaps with this dataset for 48 species. Based on museum collections, we confirmed brooding in 13 species classified as ovoviviparous by the Japanese team (*Euphaedusa subaculus*, *E. tau*, *E. stearnsii*, *E. digonoptyx*, *E. digonoptyx comes*, *Metazaptyx hachijoensis*, *Phaedusa stereoma*, *P. sieboldtii*, *Reinia echo*, *R. hungerfordiana*, *R. monelasmus*, *R. euholostoma*, *R. variegata*). On the other hand, our museum specimens of four species classified as ovoviviparous^15^ (*Phaedusa neniopsis*, *Hemiphaedusa kanaganensis*, *Reinia holotrema*, *Zaptyx hyperoptyx*) contained no embryos. It should be understood that the absence of embryos is not always informative. Soft parts may have been intentionally removed by shell collectors^55^, or snails may have been sampled during an unfavourable season^25,28^; these factors may explain false negatives in our results. In contrast, the occurrence of shelled embryos in the parental shell always proves the use of the brooding reproductive strategy. We found embryos or egg shells retained in 7 species (*Stereozaptyx exulans*, *Parazaptyx thaumatopoma*, *Metazaptyx daemonorum*, *M. pattalus*, *Zaptyx dolichoptyx*, *Z. hirasei*, and *Z. kikaiensis*) previously listed as oviparous^15^. Among these, individuals of *P. thaumatopoma*, *M. daemonorum*, and *M. pattalus* contained embryos with 1.5–2 whorls, which is usually indicative of true viviparous species. The brooding reproductive strategy of these three species was confirmed by Rei Ueshima (personal communication). Other species are likely to retain fertilised eggs only for a short time; their reproductive strategy is of an intermediate character. For the majority of old museum shell collections, information on the month of sampling was missing. Thus, we can draw no conclusions on the duration of the reproductive period in the embryo-retaining clausiliids of East and Southeast Asia, nor can we compare it with previous work on European clausiliids^23–25^. These questions can be addressed in research projects based on regular field sampling.

**Figure 6:**
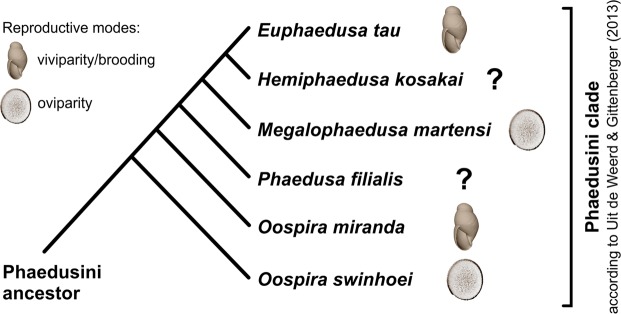
The maximum clade credibility tree for the Phaedusini lineage^49^ with reproductive modes verified in this paper. Note that the branch separating *Oospira swinhoei* from *O. miranda* is very poorly supported (Bayesian posterior probability <0.50; ML bootstrap value <50%); see Discussion.

When data on viviparous or embryo-retaining taxa were plotted on the available molecular tree of Phaedusini^49^, it appeared that brooding must have evolved more than once in this tribe. The cited authors suggested that *Euphaedusa* (brooding) forms a clade with *Megalophaedusa martensi* (oviparous), from which *Oospira miranda* (brooding) and subsequently *O. swinhoei* (oviparous) branch off basally (Fig. 6). The cited paper did not resolve the relationships between *O. miranda*, *O. swinhoei*, and the remaining taxa^49^. It was also suggested that *Oospira*, as defined by Nordsieck^46^, is not monophyletic^15^. Phylogenetic analysis covering more Phaedusini taxa is needed in order to test the hypothesis on the repeated evolution of reproductive modes in this group. Concerning the tribes Serrulinini and Synprosphymini, no evidence for brooding was found in X-rayed shells, with the exception of an egg-like structure in *Serrulina sieversi*. The number of scanned individuals from these tribes and the records in the literature concerning their reproductive biology^34,37^ were too limited for the purpose of drawing a final conclusion. Moreover, Synprosphymini were not included in the cited molecular analysis^49^. A multilocus molecular phylogeny of Phaedusinae, based on sampling of a much wider range of taxa, is currently being prepared by the authors of the present paper.

The number of embryos found in the reproductive tract in Phaedusini was usually small and reached maxima of six for *Phaedusa corticina*, eleven for *P. paviei* and *Euphaedusa tau*^31,32,36,38,39,56–58^. The number of embryos found in X-rayed shells is in accord with the data from the literature given above. A single brood kept in the genital tract of some European clausiliids may contain even twice as many embryos^14,23,29^.

It has been widely accepted that the loss or significant reduction of apertural barriers in some clausiliids (e.g. in *Balea perversa*, *Reinia variegata*, and *Macroptychia africana*) is an adaptation to viviparous reproduction^34,36,59^. As a trade-off, the apertural barriers of these species ceased to perform a protective function. However, viviparity and embryo retention also occur among species with well-developed apertural barriers in several genera and subgenera occurring in Europe, namely among *Alinda*, *Vestia*, *Pseudalinda*, *Cochlodina*, *Ruthenica*, and *Idyla*^14,23,28,30,60^. Recent biometric studies based on X-ray microcomputed tomography demonstrated the existence of adaptive changes in apertural barriers in these taxa which involved the widening of the shell channel^41^. This suggests that the greater patency of the shell channel and aperture in viviparous clausiliids must have evolved as a convergence in taxonomically distant species to enable the delivery of shelled embryos^41^. Consequently, the patency of the shell channel (and the shape of the clausilium plate which mirrors its shape) should not be of taxonomical significance. Similarly, other shell characters, such as a neck keel and a clausilium with a calcar, originated via parallel evolution in several groups of Clausiliidae^13^.

The evolution of brooding reproductive adaptation within clausiliids requires further investigation, including analysis of molecular phylogeny and in-depth histological study of the reproductive tract and pattern of embryo provision. We have shown that micro-CT screening of museum collections is a reliable method which may yield the necessary data and enhance our ability to select species for further research on reproductive strategies in land snails.

## Conclusion

Micro-CT screening of shell collections is an efficient method for testing evolutionary hypotheses concerning reproductive diversity in gastropods. We found that 1) the number of species characterised by viviparity or embryo retention in highly diversified clades of terrestrial pulmonates had been underestimated, and 2) clausiliid taxonomy based on shell characters related to viviparous reproduction should be carefully revised.

## Supplementary information

Table S1.

## Data Availability

The datasets analysed during the current study are available from the corresponding author on reasonable request.
